# Supplementary material for: KYMASIN UP Natural Product Inhibits Osteoclastogenesis and Improves Osteoblast Activity by Modulating Src and p38 MAPK
Source: Nutrients. 2022 Jul 25;14(15):3053. doi: 10.3390/nu14153053 (PMC9370798; doi:10.3390/nu14153053)
Supplement: Supplementary file 1 [file nutrients-14-03053-s001.zip › nutrients-1830667-supplementary.pdf]

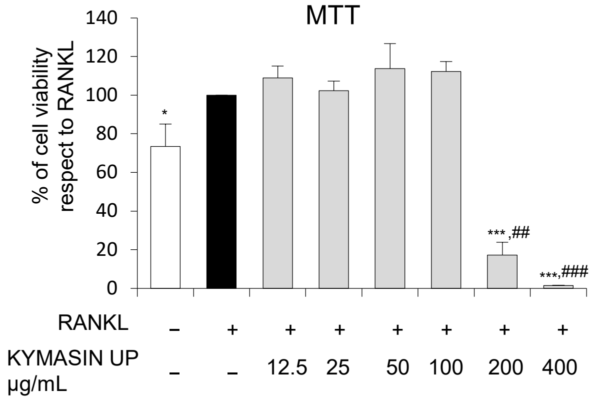

Figure S1. MTT assay. Cell viability of RAW 264.7 cells treated with RANKL for 5 days without or with KYMASIN UP (12.5-400 μg/mL) was evaluated by MTT (3-(4,5-dimethylthiazol-2-yl)-2,5-diphenyltetrazolium bromide) assay. Results are means ± standard error of the mean. Six independent experiments were performed. Statistical analysis was conducted using the two-tailed t-test. * $p < 0.05$, and *** $p < 0.001$, significantly different from RANKL. ## $p < 0.01$, and ### $p < 0.001$, significantly different from untreated control.

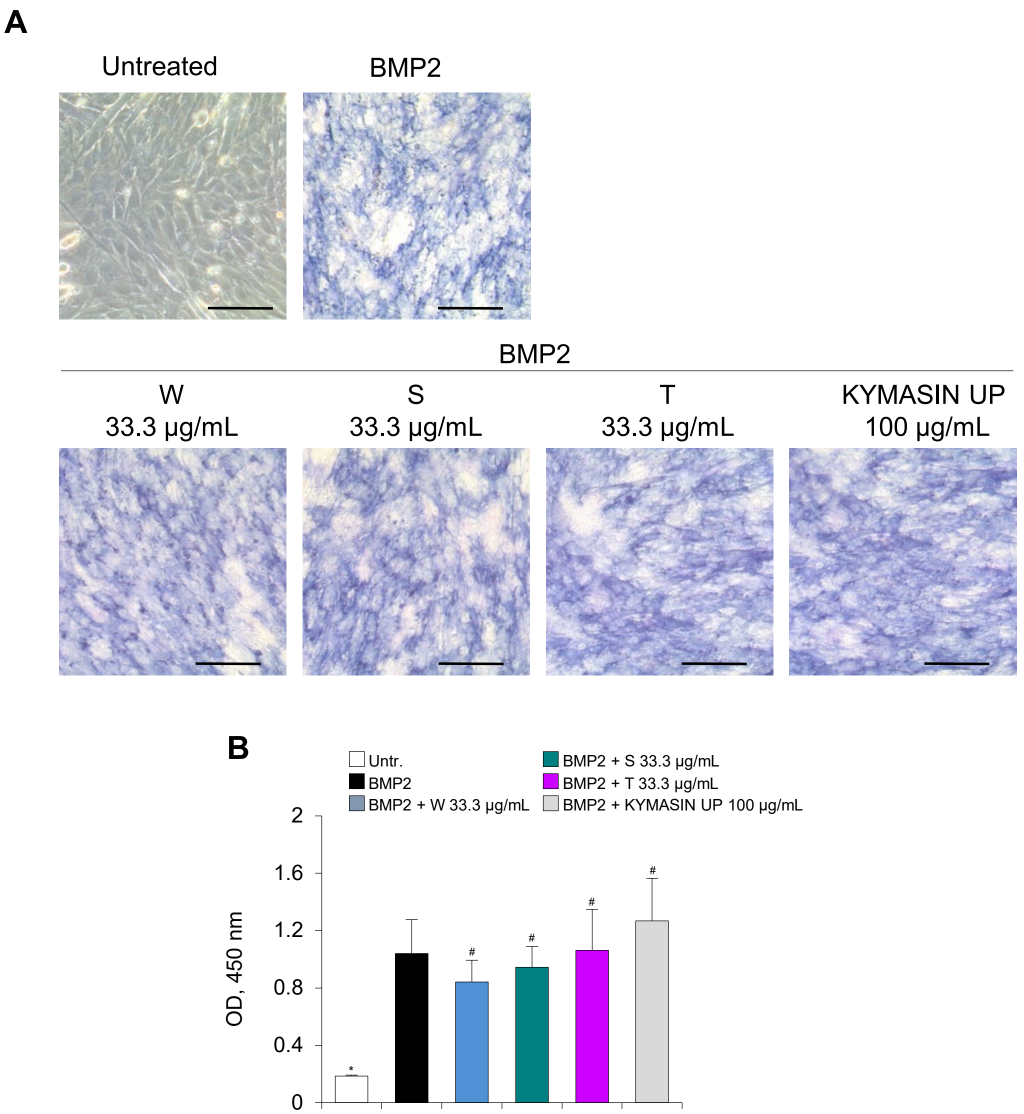

Figure S2. ALP staining and activity in C2C12 treated with single ingredients and KYMASIN UP. (A,B) C2C12 cells were treated or not with BMP2 in the absence or presence of W, S, T or KYMASIN UP at the indicated concentrations for 6 days. ALP staining was performed (A) and ALP activity was evaluated (B). Results are means ± standard deviation. Three independent experiments were performed. Statistical analysis was conducted using the two-tailed t-test. * $p < 0.05$, significantly different from BMP2. # $p < 0.05$, significantly different from untreated control (Untr). Reported are representative images (A). Scale bars (A), 200 µm.

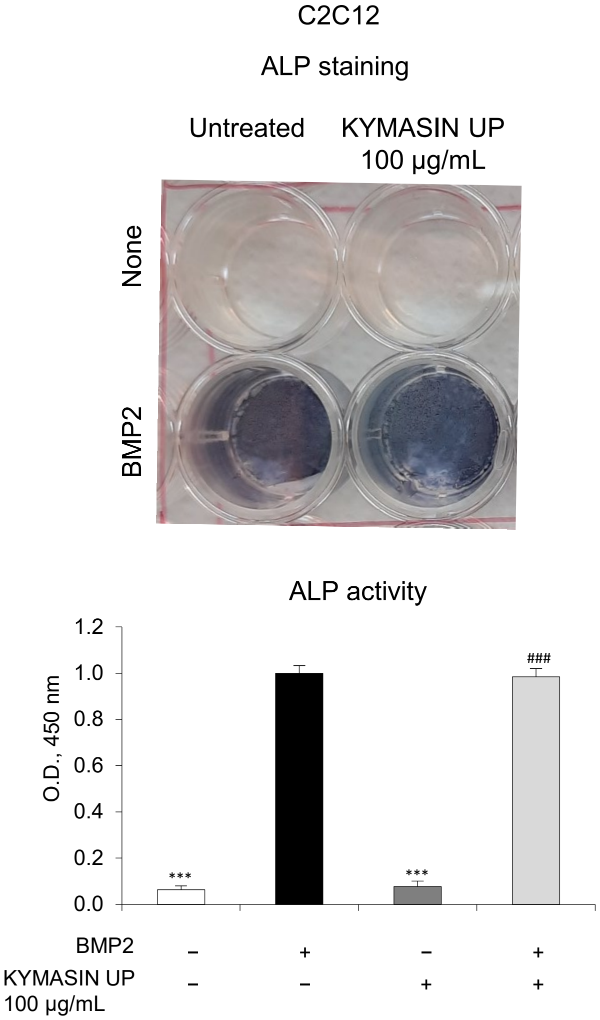

Figure S3. ALP staining and activity in C2C12 cells treated with KYMASIN UP. C2C12 cells were treated with or without BMP2 and/or KYMASIN UP (100 μg/mL) for 6 days. ALP staining was performed (upper panel) and ALP activity was measured (lower panel). Reported are representative images. Results are means ± standard deviation. Three independent experiments were performed. Statistical analysis was conducted using the two-tailed t-test. *** $p < 0.001$, significantly different from BMP2. ### $p < 0.001$, significantly different from KYMASIN UP.

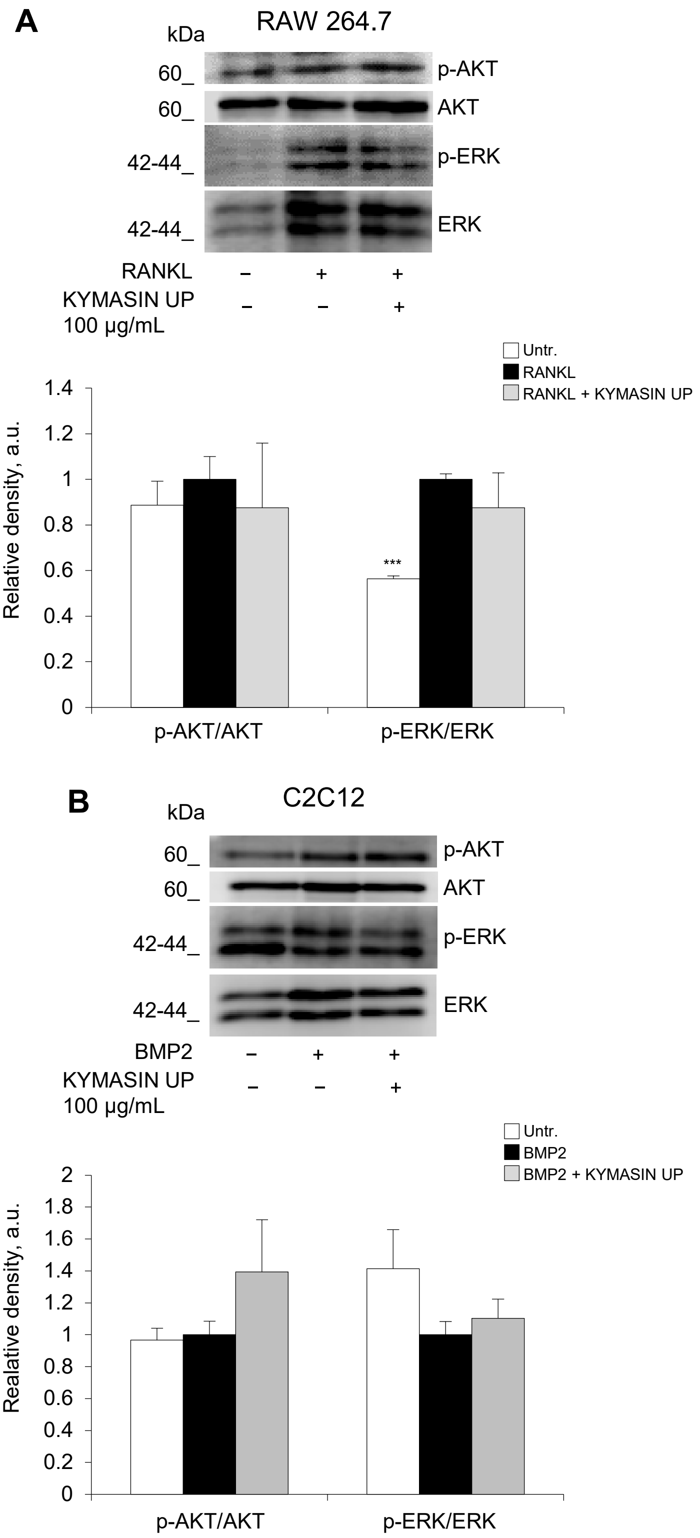

Figure S4. ERK1/2 and AKT pathways activation state in the presence of KYMASIN UP. (A,B) The expressions of phosphorylated (p) AKT (protein kinase B) and ERK1/2 (extracellular signal-regulated kinases) were analyzed by western blotting in RAW 264.7 cells treated with RANKL for 5 days (A) or in C2C12 cells treated with BMP2 for 6 days (B), in the absence or presence of KYMASIN UP (100 µg/mL). Reported are representative images and the relative densities with respect to the total forms of AKT and ERK1/2. Results are means ± standard error of the mean. Six independent experiments were performed. Statistical analysis was conducted using the two-tailed t-test. *** $p < 0.001$, significantly different from RANKL.
